# Handling missing data in RCTs; a review of the top medical journals

**DOI:** 10.1186/1471-2288-14-118

**Published:** 2014-11-19

**Authors:** Melanie L Bell, Mallorie Fiero, Nicholas J Horton, Chiu-Hsieh Hsu

**Affiliations:** Division of Epidemiology and Biostatistics, Mel and Enid Zuckerman College of Public Health, University of Arizona, Tucson, AZ 85724 USA; Department of Mathematics and Statistics, Amherst College, Amherst, MA 01002 USA

**Keywords:** Missing data, Intention-to-treat, Sensitivity analysis

## Abstract

**Background:**

Missing outcome data is a threat to the validity of treatment effect estimates in randomized controlled trials. We aimed to evaluate the extent, handling, and sensitivity analysis of missing data and intention-to-treat (ITT) analysis of randomized controlled trials (RCTs) in top tier medical journals, and compare our findings with previous reviews related to missing data and ITT in RCTs.

**Methods:**

Review of RCTs published between July and December 2013 in the *BMJ*, *JAMA*, *Lancet*, and *New England Journal of Medicine*, excluding cluster randomized trials and trials whose primary outcome was survival.

**Results:**

Of the 77 identified eligible articles, 73 (95%) reported some missing outcome data. The median percentage of participants with a missing outcome was 9% (range 0 – 70%). The most commonly used method to handle missing data in the primary analysis was complete case analysis (33, 45%), while 20 (27%) performed simple imputation, 15 (19%) used model based methods, and 6 (8%) used multiple imputation. 27 (35%) trials with missing data reported a sensitivity analysis. However, most did not alter the assumptions of missing data from the primary analysis. Reports of ITT or modified ITT were found in 52 (85%) trials, with 21 (40%) of them including all randomized participants. A comparison to a review of trials reported in 2001 showed that missing data rates and approaches are similar, but the use of the term ITT has increased, as has the report of sensitivity analysis.

**Conclusions:**

Missing outcome data continues to be a common problem in RCTs. Definitions of the ITT approach remain inconsistent across trials. A large gap is apparent between statistical methods research related to missing data and use of these methods in application settings, including RCTs in top medical journals.

**Electronic supplementary material:**

The online version of this article (doi:10.1186/1471-2288-14-118) contains supplementary material, which is available to authorized users.

## Background

While randomized controlled trials are considered to be the gold standard of intervention research in the biomedical setting, their validity can be threatened by missing outcome data. Participants with missing data are often a non-random subset of the sample, increasing the risk of biased estimates of treatment effects. The intention to treat (ITT) principle, in short, “analyze as randomized”, is recognized as an important protection against bias by preserving the benefits of randomisation--namely balancing both known and unknown factors and eliminating selection bias [1, 2]. When outcome data are missing, however, a true ITT analysis can be difficult or impossible to achieve, and researchers must make assumptions, some of which may be strong and unverifiable [3, 4]. In the presence of incomplete data therefore, it is imperative to perform sensitivity analyses, which examine the robustness of the results to assumptions made in the primary analysis [5–8].

Missing data can reduce the power and efficiency of a study but, unfortunately, can also lead to biased results [5–7]. For example, if patients experiencing high toxicity are more likely to drop out of the trial, quality of life is likely to be overestimated and toxicity underestimated. Missing data and statistical approaches for handling them have been an active area of research and the following definitions are commonly used [9]. If missingness of the outcome of interest is unrelated to observed or unobserved patient data, the missing data are termed missing completely at random (MCAR): a strong assumption. If data are MCAR, analyzing only those with observed outcome data (complete case analysis) will result in some loss of efficiency but unbiased estimation [5–7]. If after taking observed data into account there are no systematic differences between participants with complete data as compared to those with missing data , data are considered to be missing at random (MAR). Multiple imputation [10] and model-based approaches, such as mixed models [11] and weighted generalized estimating equations (GEE) [12] for repeatedly measured outcomes, based on all observed data can be valid and unbiased methods for MAR data, as long as the models are specified correctly . Missing outcomes are termed missing not at random (MNAR) if systematic differences between dropouts and completers persist even after taking observed data into account. The once popular simple imputation approach of replacing missing data with the last observation carried forward (LOCF) (for longitudinal outcome data) is not necessarily valid under any of these missingness assumptions [13, 14]. Sensitivity analyses should be performed under different assumptions than the primary analysis for example, if the primary analysis makes a MCAR assumption, the sensitivity analyses should assume MAR or MNAR.

The statistical literature is rich with methods for handling incomplete data, including approaches for sensitivity analysis and MNAR data. Guidelines for handling missing data have been published in journals such as the Journal of Clinical Oncology [15], the BMJ [4, 16, 17] and the New England Journal of Medicine [18]. The widely adopted CONSORT statement includes a set of checklists on ITT and missing data [19]. Researchers in the field of missing data, including the Panel on Handling Missing Data in Clinical Trials commissioned recently by the National Research Council, have made calls for the use of improved methods for handling missing data including sensitivity analyses, and for more rigorous approaches to ITT analysis when outcome data are missing [4–7, 16, 20].

Reviews of missing data and ITT in RCTs published in top medical journals for the years 1997, 2001, 2002 and 2005-2006 have been carried out [1, 2, 21, 22]. These reviews concluded that missing outcome data in RCTs are widespread; poor handling of missing data is the norm; the term ITT is common but inconsistently used; and sensitivity analyses are rarely (if ever) reported. With the recent guidelines and exhortations to more appropriately handle missing data (including planning and prevention), we hypothesized that the amount of missing data would have decreased and current approaches would have improved. The aims of this study, therefore, were to identify, in RCTs published in the top medical journals, the proportion of trials:

1. with missing data and their handling of missing data;
2. reporting sensitivity analyses regarding missing data; and
3. reporting an intention to treat analysis.

Secondary aims included assessing indicators of planning for and prevention of, missing outcome data, and to compare current approaches with those reported in the previous reviews.

## Methods

We performed a PubMed search of randomized controlled trials published in four top medical journals: the *British Medical Journal* (BMJ); *Journal of the American Medical Association* (JAMA); *The Lancet*; and *New England Journal of Medicine* (NEJM) between July and December 2013. Cluster randomized trials and trials whose primary outcomes were survival were excluded because 1) the statistical issues for these are different to those in individually randomized and for non-survival outcomes and 2) we wanted to compare our results to a previous review (see below). The search strategy included searching for studies in each journal whose publication type was classified as “randomized controlled trials”. We examined each paper collected from the initial search and identified relevant studies based on study exclusion criteria.

### Content assessment

All articles were assessed by one reviewer (MF) using a standardized form, and 15% of the studies were randomly selected to be independently assessed by a second and third reviewer. We calculated kappa statistics to evaluate inter-rater reliability for methods used to handle missing data in primary analysis, whether an intention-to-treat analysis was performed, and sensitivity analysis. All disagreements were resolved by consensus.

#### Extent and handling of missing data

For each article we determined the magnitude of missing data and method(s) for handling missing data in the principal analysis. We defined the number of subjects per trial as the number of subjects randomized. The proportion with a missing outcome was computed as the number of subjects with a missing outcome divided by the total number of subjects randomized. The principal analysis was defined as the main analysis performed on the primary outcome. When more than one primary outcome was reported in the trial, we used the outcome that appeared first in the methods section. For primary outcome measurements monitored repeatedly, we used the final follow-up time point to calculate the missing rate, unless a preceding time point was specified. We identified the statistical method used to handle missing data in the principal analysis and classified these as complete case, simple imputation (such as last or worst observation carried forward), multiple imputation or model based (for example, mixed models or generalized estimating equations). Complete case was defined as using only individuals who had complete primary outcome data for the stated primary analysis. To assess prevention and planning, we recorded whether mention was made of attempts to avoid missing data, whether sample size calculations accounted for missing data, and by how much observed and expected attrition rates differed.

#### Sensitivity analysis for missing data

We assessed method(s) to deal with missing data in any sensitivity analysis and calculated the proportion of trials that reported carrying out a sensitivity analysis. We defined sensitivity analysis as any alternative technique performed to further investigate the effects of missing outcome data on primary results.

#### Intention-to-treat analysis

We determined the proportion of trials that reported an ITT or modified ITT analysis, and verified whether all randomized subjects were analyzed and how missing data were handled if any.

### Comparison of reviews on missing data

We compared our findings with previous reviews of missing data and ITT in RCTs. For each review we reported, if possible, the number of trials: included in the review, with missing data, reporting sensitivity analysis, reporting ITT, and missing data approaches. We aimed to compare our results directly to Wood et al, [16] to assess whether changes have occurred since their 2004 paper on RCTs published in 2001. We therefore used similar definitions, inclusion criteria and collected data in a similar fashion. We used chi-square tests for comparisons. Other reviews were compared qualitatively.

## Results

Our search identified 148 randomized controlled trials published within the six-month period. A total of 71 trials were excluded (19 were cluster randomized controlled trials, 52 had a primary outcome as time to event), leaving 77 articles to review. Inter-rater agreement for methods used to handle missing data in primary analysis, intention-to-treat, and sensitivity analysis were 0.72, 0.94, and 0.78, respectively, showing “substantial” to “near perfect” agreement, according to Landis and Koch [23]. Table 1 shows the general characteristics of the included trials. Together, the median number of subjects randomized was 368, with a range of 13 – 53,450. A list of included studies can be found in Additional file 1.

**Table 1:** **General characteristics of the 77 randomized controlled trials published July – December 2013**

	N (%)
Journal	
BMJ	8 (10)
JAMA	26 (34)
Lancet	22 (29)
NEJM	21 (27)
Number of centers involved	
Single	21 (27)
Multiple	56 (73)
Type of outcome	
Quantitative	42 (55)
Binary	35 (45)
How often outcome was collected	
Single	16 (21)
Repeated	61 (79)
How outcome was treated in the primary analysis	
Single	63 (82)
Repeated	14 (18)
Reported CONSORT flow diagram	76 (99)
Reported primary analysis was intention-to-treat or modified intention-to-treat	66 (86)

### Extent and handling of missing data

Seventy-three (95%) trials reported some missing outcome data. The median percentage of patients with a missing outcome was 9%, with a range of 0 – 70%. Sixty-six trials reported reasons why outcomes were missing with reasons ranging from simply stating that patients were lost to follow-up to very specific explanations. The majority of trials reported these details in their CONSORT flow diagram [19].

Complete case analysis was the most common method to handle missing data (33, 45%). Twenty (27%) trials used simple imputation methods. Three (5%) used linear interpolation, eight (11%) used worst-case imputation, nine (12%) used last observation carried forward. Six (8%) trials performed multiple imputation. Fifteen (19%) trials used model based methods: 11 (15%) used mixed models and three (4%) used un-weighted generalized estimating equations (Table 2). Thus, a MAR assumption for the primary analysis was made in 17 (23%) of the trials with missing data.

**Table 2:** **Handling of missing data in primary analysis among 73 trials who reported missing outcome data**

	N (%) ^1^
Proportion of patients with missing outcome^1^	
No missing data	4 (5)
0 – 1%	2 (3)
1 – 5%	11 (14)
5 – 10%	24 (31)
> 10%	36 (47)
Reported number of patients with missing outcomes by randomized treatment arm	71 (97)
Reported reasons why missing	66 (90)
Mentioned attempts to avoid missing data before and during trial	26 (36)
Methods	
Complete case	33 (45)
Simple imputation	
Linear interpolation	3 (5)
Worst-case	8 (11)
LOCF	9 (12)
Multiple imputation	6 (8)
Model based	
GEE (un-weighted)	3 (4)
Mixed model/hierarchical/multilevel	11 (15)

^1^The denominator for the proportion of patients with missing outcome is 77; the other denominators are 73 (the number of studies with any missing data).

Of the trials reporting more than 10% missing data, 13 (36%) used complete case, 13 (36%) used simple imputation, 3 (8%) used multiple imputation, and 7 (19%) used model based methods. Of the trials reporting less than 10% missing data, 20 (54%) used complete case, 7 (19%) used simple imputation, 3 (8%) used multiple imputation, and 7 (19%) used model based methods.

Sixty-six (86%) trials presented a sample size calculation, with 38 of them accounting for missing data in the calculation by inflating the sample size by one minus the expected attrition rate. The mean absolute difference in the actual attrition rate and the expected was 8% with a range of 0.3–31%. Two trials accounting for missing data in the sample size calculation had unclear expected attrition rates. 72% estimated higher attrition rates than observed, while 28% estimated lower attrition rates than observed.

Attempts to avoid missing data before and during the trial were mentioned in 26 trials. The median percentage of missing data for those who mentioned attempts to avoid missing data was 12%, with a range of 2–56%. The median percentage of missing data for those who did not mention attempts to avoid missing data was 9%, with a range of 0.6 – 70%.

Seventy-one (97%) trials reported the number of patients with missing outcome by treatment arm. Nine trials reported comparisons of baseline characteristics between patients with observed and missing outcomes. Six of them reported a significant difference.

### Sensitivity analysis for missing data

Twenty-seven trials (35%) reported performing a sensitivity analysis with respect to missing data (Table 3). Of these, ten (37%) trials used multiple imputation in the sensitivity analysis. Six (22%) performed a complete case analysis. Four (15%) trials carried out simple imputation: two performed worst-case imputation and one imputed with baseline value. One trial performed both complete case analysis and LOCF. One trial performed worst-case imputation, LOCF, and multiple imputation. Two trials carried out adjustments using auxiliary data. One trial used un-weighted GEE and two trials used mixed models. Two trials reported that they performed a sensitivity analysis, but methods were unclear.

**Table 3:** **Methods for handling missing data in sensitivity analysis in 27 trials**

Sensitivity method	Assumption	Primary analysis	Assumption	N	Total N (%)
Complete case	MCAR	Simple imputation	MCAR	3	6 (22)
		MI	MAR	2	
		Mixed Model	MAR	1	
Simple imputation^1^	MCAR	GEE	MCAR	1	4 (15)
		MI	MAR	1	
		Mixed model	MAR	2	
GEE (un-weighted)	MCAR	Complete case	MCAR	1	1 (4)
MI^2^	MAR	Complete case	MCAR	6	10 (37)
		Simple imputation	MCAR	1	
		GEE	MCAR	1	
		Mixed model	MAR	2	
Mixed model	MAR	Complete case	MCAR	2	2 (7)
Adjustment using auxiliary data	MAR	Mixed model	MAR	2	2 (7)
Unclear		Complete case	MCAR	1	2 (7)
		Simple imputation	MCAR	1	

^1^One trial also performed complete case analysis.

^2^One trial also performed simple imputation.

In total, 11 made a MCAR assumption for the sensitivity analysis, and 15 made a MAR assumption. Only 10 weakened the missingness assumption of the primary analysis to perform their sensitivity analysis, by using a MCAR assumption for the primary followed by a MAR assumption in the sensitivity. No researchers used MNAR models. Of the 36 trials reporting more than 10% missing data, 16 (44%) performed a sensitivity analysis: 4 used complete case, 2 used simple imputation, 1 used simple and multiple imputation, 6 used multiple imputation, 2 made adjustments using auxiliary data, and 1 approach was unclear. Of the 41 trials reporting less than 10% missing data, 11 (27%) performed a sensitivity analysis: 2 used complete case, 1 used complete case and simple imputation, 1 used simple imputation, 3 used multiple imputation, 3 used model based methods, and 1 was unclear.

### Intention-to-treat analysis

Fifty-two reported the use of ITT and 14 reported the use of modified ITT for their primary analysis. Definitions of ITT and modified ITT differed across trials. Of the articles that reported ITT or modified ITT analysis, 21 (40%) included all randomized subjects in the primary analysis. Of the articles that reported ITT or modified ITT analysis, 62 (94%) had missing data. Of the trials with missing data, 48 (66%) reported ITT analysis and 14 (19%) reported modified ITT analysis.

### Comparison of reviews on missing data

Of the five reviews that we considered, including our own, missing data rates were fairly similar, ranging from 61–95%. Use of complete case analysis and simple imputation were consistent across reviews, ranging from 45–65% and 17–27%, respectively. Recently, there has been an increase in application of multiple imputation and model based methods for missing data in primary analyses. The number of papers reporting sensitivity analysis for missing data (1–37%) and ITT analysis (48–85%) have both increased across time. See Table 4.

**Table 4:** **Reviews on missing data and ITT in the BMJ, JAMA, NEJM and the Lancet** ^**1**^

Study	Timing	Study inclusion criteria	Number of trials included	Number (%) of papers with missing data ^2^	Number (%) of papers with more than 10% missing data ^2^	Missing data approaches in primary analysis	Number (%) ^3^	Number (%) of papers reporting sensitivity analysis ^3^	Number (%) of papers reporting ITT ^4^
Hollis et al., 1999 [2]	1997	All RCTs	249	89/119 (75)	29/119 (24)	Complete case	44 (49)	1 (1)	119/249 (48)
						Simple imputation	15 (17)		
						Multiple imputation	0		
						Model based	29 (33)		
						Unclear	1 (1)		
Wood et al., 2004 [22]	July-Dec, 2001	All RCTs with non-survival outcomes	71	63/71 (89)	36/71 (51)	Complete case	41 (65)	13 (21)	26/63 (41)
						Simple imputation	14 (22)		
						Multiple imputation	1 (2)		
						Model based	5 (8)		
						Unclear	2 (3)		
Gravel et al., 2007 [1]	2002	Sample of RCTs^1^	403	152/249 (61)	52/249 (21)	Complete case	89 (59)	Not reported	249/403 (62)
						Simple imputation	32 (21)		201/283 (71)^5^
						Multiple imputation	1 (1)		
						Model based^6^	0		
						Unclear^6^	30 (20)		
Fielding et al., 2008 [21]	2005-2006	Random sample of RCTs with Quality of life outcomes	61	55/61 (90)	22/61 (36)	Complete case	30 (55)	6 (11)	Not reported
						Simple imputation	11 (20)		
						Multiple imputation	1 (2)		
						Model based	9 (16)		
						Unclear	4 (7)		
Bell et al., (current study)	July-Dec, 2013	All RCTS with non-survival outcomes	77	73/77 (95)	36/77 (47)	Complete case	33 (45)	27 (37)	62/73 (85)
						Simple imputation	20 (27)		
						Multiple imputation	6 (8)		
						Model based	14 (19)		

^1^Gravel et al. reported on 10 journals, including the BMJ, JAMA, NEJM and the Lancet.

^2^Denominator is the number of trials included except for Hollis et al. and Gravel et al., where denominators are the number of papers reporting ITT.

^3^Denominator is the number of papers with missing data.

^4^Denominator is the number of papers with missing data except for Hollis et al. and Gravel et al. where denominators are the number of trials included in the review.

^5^Sub-analysis of RCTs from the four journals (BMJ, JAMA, NEJM and the Lancet) out of the 10 journals included in Gravel’s review.

^6^Three reported as “other” might be model based (added to 27 marked “unclear”).

In comparison to Wood et al.’s [22] review of RCTs from 2001, trials reporting missing data were similar: 89% versus 95% (95% confidence interval (CI) for difference -3% to 15%; P = 0.18). The percentage of papers with 10% or more missing data was also similar, at 51% versus 47% (95% CI for difference -12% to 20%; P = 0.63). Of those that reported missing data, trials that used complete case analysis decreased from 65% to 45% (95% CI for difference -36% to -3%; P = 0.02). The percent that performed simple imputation was similar, at 22% as compared to 27% (95% CI for difference -9% to 19%; P = 0.49). The use of multiple imputation or model based methods increased from 10% to 27% (95% CI for difference 5% to 30%; P = 0.01). Mentions of attempts to avoid missing data was similar, at 35% compared to 29% (95% CI for difference -46% to -58%; P = 0.83). Sensitivity analysis for missing data increased from 21% to 37% (95% CI for difference 1% to 31%; P = 0.04). Reports of ITT or modified ITT analysis increased from 41% to 85% (95% CI for difference 29% to 59%; P <0.0001).

## Discussion

### Summary

Our review of 77 RCTs published in the top medical journals found that 95% of trials reported some missing outcome data, with a median of 9%, and up to 70%. Complete case analysis was the most common way of handling missing data in the primary analysis (45%), followed by simple imputation (27%), model based methods (mixed models and un-weighted generalized estimating equations) (19%) and multiple imputation (8%). Sensitivity analyses were performed in 35% of the trials, but most (63%) did not weaken the assumptions regarding missing data from their primary analysis. An ITT or modified ITT was reported in 85% of the trials. Most reports included a sample size calculation (86%), and 58% of these inflated the sample size to account for expected attrition. These calculations tended to be conservative, with 72% estimating higher dropout than observed with a difference of 8%, and ranging up to 30% higher.

### Relation to other literature

The amount of missing data appears to have remained fairly constant over time, as does the proportion of trials that mentioned attempts to avoid missing data. While it is possible that those trials that did not report prevention attempts did, in fact, employ them, it may be that researchers need to give more consideration to missing data during trial design and conduct.

The use of methods with the strong assumption that data are missing completely at random (complete case analysis, simple imputation and un-weighted GEE) has remained popular: 85% in the current review as compared to 89% in the 2001 review. This is in direct contrast to recommendations put forth by leaders in the field, including the National Research Council’s Committee on National Statistics (CNSTAT) Panel on Handling Missing Data in Clinical Trials which recommends a primary analysis that assumes data are missing at random, followed by sensitivity analyses which weaken this assumption and allows for data not missing at random [7, 18]. White et al. [4] suggest a four part strategy: 1) attempt to follow up all subjects; 2) carry out a primary analysis of all observed data that are valid under a plausible assumption; 3) perform sensitivity analyses to explore the effect of departures from the primary assumption; and 4) account for all randomized participants in at least one of the analyses. This approach was utilized in the analysis of an alcohol screening and brief intervention study [24]. It was discouraging that sensitivity analyses which contradict the assumptions of the primary analyses remain so rare.

The CNSTAT report favored inverse probability weighted generalized estimating equation (GEE) methods and multiple imputation, in part because auxiliary data associated with missingness can be incorporated into the analysis [18]. However, none of the RCTs in our review used weighted GEEs, and only 8 used MI for the primary analysis. While the number of trials reporting sensitivity analyses appear to have increased over time, from 1% in 1997, 21% in 2001, and up to 37% in our review, none of the studies reported using MNAR models, or appeared to follow the Panel’s guidelines for sensitivity analyses. Perhaps the reluctance to use more sophisticated approaches is due to a lack of knowledge or experience on the parts of applied researchers and/or biostatisticians. Perhaps it is due to the time lag between reports of methods and software to implement them.

Reports of ITT or modified ITT analysis have increased substantially over time, from 48% in 1997, 41% in 2001, 71% in 2007, to 85% in 2013. However, we found, as others have [1, 2], that these terms are used inconsistently: only 40% actually included all randomized participants in the primary analysis. White et al. [20] call for at least one analysis (primary or sensitivity) to include all participants. This lack of consistent definition (even amongst methodologists [25]) and clarity regarding who was included in the analysis has led the CONSORT statement’s authors to remove the ITT request in their 2010 update (over the original 2001 statement) [19].

Many trials had repeated measurements, (79%) but only 14 of these used all the measurements in the primary analysis, often resulting in a strong MCAR assumption. Using all outcome data, even if the primary interest is in a specific time point, can reduce some or all of the bias due to data which are missing non-randomly. For example, Bell and Fairclough use several methods to analyze quality of life measured at four time points in an RCT with substantial missing data. A t-test comparing the two arms at the 4^th^ timepoint found a treatment effect of -0.4; a contrast from a mixed model estimated the effect to be -8.0 [5]. Simulation studies have demonstrated the bias that can occur when a MCAR assumption is made for data which are MAR [17, 26]. Approaches which use all repeated measures data and are valid for MAR data include multiple imputation, mixed models, inverse probability weighted GEEs, and Bayesian analysis [5–7].

### Strengths and limitations

A strength of our review is the inclusion of other reviews to assess possible time trends. In particular, we followed Wood and colleagues [22] methods and definitions in order to make direct comparisons between 2001 and 2013. A limitation is the difficulty in making comparisons with the other reviews, due to different inclusion criteria. For example, inclusion of survival outcomes may reduce missing data rates, as participants who drop out are often considered censored. Different definitions may also hinder comparisons. For example, the rate of missing data when measurements are taken repeatedly could be: 1) the number of patients with any missing primary outcome data divided by the total number of patients randomized; or 2) the number of missing assessments divided by the total number of assessments. We chose the former, in line with Wood et al. [22] Another limitation is that we focused on the top 4 medical journals. It is likely that trial reports appearing in these journals have higher standards of conduct and reporting, so that this review may underestimate the extent of missing data and overestimate the use of sensitivity analyses.

### Recommendations and conclusion

We have several recommendations. First, missing data should be considered at each stage of a trial: design, conduct, analysis, and reporting. Prevention is the best way to handle missing data, so more effort needs to be put into missing data at the design and conduct stage. The CNSTAT report [7], and it’s synopsis [18] discuss several approaches. Second, we recommend that in trials with repeated measurements, all data should be used in an analysis that makes a plausible assumption about missing data. Usually this will be a MAR assumption. Third, sensitivity analyses that weaken the assumptions about missing data should be carried out and reported. For example, if the primary analysis uses a MAR assumption, the sensitivity analysis should assume MNAR.

There appears to be a large gap in translation between statistical methods research and the use of these methods in applications, such as RCTs. For example, simple imputation remains popular, despite warnings from many statisticians against their use, particularly LOCF [5–7, 13, 14, 27–29]. This failure to translate persists, despite papers regarding missing data, sensitivity analyses, and strategies for intention to treat in the presence of missing data being published in high impact medical journals [4, 16–18]. More statisticians should attempt to make their work accessible to applied researchers, by publishing secondary papers in appropriate applied journals showing how to make their methods work in practice. Applied statisticians and researchers should read these papers to update their skillsets and use modern methods that increase statistical power and in some cases reduce bias. Editors and reviewers should demand that modern methods which use all the data are used, at least in the sensitivity analysis.

## Conclusions

Applied researchers and statisticians need to improve their handling of missing data in RCTs.

## Authors’ information

Melanie L Bell is Associate Professor of Biostatistics. Mallorie Fiero is a PhD candidate in Biostatistics. Nicholas J Horton is Professor of Biostatistics. Chiu-Hsieh Hsu is Associate Professor of Biostatistics.

## Electronic supplementary material

Additional file 1: **References of the 77 trials included in the missing data in RCTs review.** (DOCX 36 KB)

## Abbreviations

ITT: Intention to treat
MCAR: Missing completely at random
MAR: Missing at random
MNAR: Missing not at random
GEE: Generalized estimating equations
LOCF: Last observation carried forward
RCT: Randomized controlled trial
MI: Multiple imputation
CI: Confidence interval.
