# Supplementary material for: Handling missing data in RCTs; a review of the top medical journals
Source: BMC Med Res Methodol. 2014 Nov 19;14:118. doi: 10.1186/1471-2288-14-118 (PMC4247714; doi:10.1186/1471-2288-14-118)
Supplement: Supplementary file 1 — Additional file 1: References of the 77 trials included in the missing data in RCTs review. (DOCX 36 KB) [file 12874_2014_1131_MOESM1_ESM.docx]

**Additional File 1**

Below are the references of the 77 trials included in the missing data in RCTs review.

1. Abalos E, Addo V, Brocklehurst P, El Sheikh M, Farrell B, Gray S, Hardy P, Juszczak E, Mathews JE, Masood SN *et al*: **Caesarean section surgical techniques (CORONIS): a fractional, factorial, unmasked, randomised controlled trial**. *Lancet* 2013, **382**(9888):234-248.

2. Abdallah MS, Wang K, Magnuson EA, Spertus JA, Farkouh ME, Fuster V, Cohen DJ: **Quality of life after PCI vs CABG among patients with diabetes and multivessel coronary artery disease: a randomized clinical trial**. *Jama* 2013, **310**(15):1581-1590.

3. Abed HS, Wittert GA, Leong DP, Shirazi MG, Bahrami B, Middeldorp ME, Lorimer MF, Lau DH, Antic NA, Brooks AG *et al*: **Effect of weight reduction and cardiometabolic risk factor management on symptom burden and severity in patients with atrial fibrillation: a randomized clinical trial**. *Jama* 2013, **310**(19):2050-2060.

4. Aberle DR, DeMello S, Berg CD, Black WC, Brewer B, Church TR, Clingan KL, Duan F, Fagerstrom RM, Gareen IF *et al*: **Results of the two incidence screenings in the National Lung Screening Trial**. *N Engl J Med* 2013, **369**(10):920-931.

5. Allen SJ, Wareham K, Wang D, Bradley C, Hutchings H, Harris W, Dhar A, Brown H, Foden A, Gravenor MB *et al*: **Lactobacilli and bifidobacteria in the prevention of antibiotic-associated diarrhoea and Clostridium difficile diarrhoea in older inpatients (PLACIDE): a randomised, double-blind, placebo-controlled, multicentre trial**. *Lancet* 2013, **382**(9900):1249-1257.

6. Andreyev HJ, Benton BE, Lalji A, Norton C, Mohammed K, Gage H, Pennert K, Lindsay JO: **Algorithm-based management of patients with gastrointestinal symptoms in patients after pelvic radiation treatment (ORBIT): a randomised controlled trial**. *Lancet* 2013, **382**(9910):2084-2092.

7. Baeten D, Baraliakos X, Braun J, Sieper J, Emery P, van der Heijde D, McInnes I, van Laar JM, Landewe R, Wordsworth P *et al*: **Anti-interleukin-17A monoclonal antibody secukinumab in treatment of ankylosing spondylitis: a randomised, double-blind, placebo-controlled trial**. *Lancet* 2013, **382**(9906):1705-1713.

8. Barnett AH, Huisman H, Jones R, von Eynatten M, Patel S, Woerle HJ: **Linagliptin for patients aged 70 years or older with type 2 diabetes inadequately controlled with common antidiabetes treatments: a randomised, double-blind, placebo-controlled trial**. *Lancet* 2013, **382**(9902):1413-1423.

9. Barrett JF, Hannah ME, Hutton EK, Willan AR, Allen AC, Armson BA, Gafni A, Joseph KS, Mason D, Ohlsson A *et al*: **A randomized trial of planned cesarean or vaginal delivery for twin pregnancy**. *N Engl J Med* 2013, **369**(14):1295-1305.

10. Bergenstal RM, Klonoff DC, Garg SK, Bode BW, Meredith M, Slover RH, Ahmann AJ, Welsh JB, Lee SW, Kaufman FR: **Threshold-based insulin-pump interruption for reduction of hypoglycemia**. *N Engl J Med* 2013, **369**(3):224-232.

11. Berk JL, Suhr OB, Obici L, Sekijima Y, Zeldenrust SR, Yamashita T, Heneghan MA, Gorevic PD, Litchy WJ, Wiesman JF *et al*: **Repurposing diflunisal for familial amyloid polyneuropathy: a randomized clinical trial**. *Jama* 2013, **310**(24):2658-2667.

12. Bishop N, Adami S, Ahmed SF, Anton J, Arundel P, Burren CP, Devogelaer JP, Hangartner T, Hosszu E, Lane JM *et al*: **Risedronate in children with osteogenesis imperfecta: a randomised, double-blind, placebo-controlled trial**. *Lancet* 2013, **382**(9902):1424-1432.

13. Bullen C, Howe C, Laugesen M, McRobbie H, Parag V, Williman J, Walker N: **Electronic cigarettes for smoking cessation: a randomised controlled trial**. *Lancet* 2013, **382**(9905):1629-1637.

14. Cahn P, Pozniak AL, Mingrone H, Shuldyakov A, Brites C, Andrade-Villanueva JF, Richmond G, Buendia CB, Fourie J, Ramgopal M *et al*: **Dolutegravir versus raltegravir in antiretroviral-experienced, integrase-inhibitor-naive adults with HIV: week 48 results from the randomised, double-blind, non-inferiority SAILING study**. *Lancet* 2013, **382**(9893):700-708.

15. Caroli A, Perico N, Perna A, Antiga L, Brambilla P, Pisani A, Visciano B, Imbriaco M, Messa P, Cerutti R *et al*: **Effect of longacting somatostatin analogue on kidney and cyst growth in autosomal dominant polycystic kidney disease (ALADIN): a randomised, placebo-controlled, multicentre trial**. *Lancet* 2013, **382**(9903):1485-1495.

16. Cefalu WT, Leiter LA, Yoon KH, Arias P, Niskanen L, Xie J, Balis DA, Canovatchel W, Meininger G: **Efficacy and safety of canagliflozin versus glimepiride in patients with type 2 diabetes inadequately controlled with metformin (CANTATA-SU): 52 week results from a randomised, double-blind, phase 3 non-inferiority trial**. *Lancet* 2013, **382**(9896):941-950.

17. Chakravarthy U, Harding SP, Rogers CA, Downes SM, Lotery AJ, Culliford LA, Reeves BC: **Alternative treatments to inhibit VEGF in age-related choroidal neovascularisation: 2-year findings of the IVAN randomised controlled trial**. *Lancet* 2013, **382**(9900):1258-1267.

18. Chen HH, Anstrom KJ, Givertz MM, Stevenson LW, Semigran MJ, Goldsmith SR, Bart BA, Bull DA, Stehlik J, LeWinter MM *et al*: **Low-dose dopamine or low-dose nesiritide in acute heart failure with renal dysfunction: the ROSE acute heart failure randomized trial**. *Jama* 2013, **310**(23):2533-2543.

19. Chesterton LS, Lewis AM, Sim J, Mallen CD, Mason EE, Hay EM, van der Windt DA: **Transcutaneous electrical nerve stimulation as adjunct to primary care management for tennis elbow: pragmatic randomised controlled trial (TATE trial)**. *Bmj* 2013, **347**:f5160.

20. Doody RS, Raman R, Farlow M, Iwatsubo T, Vellas B, Joffe S, Kieburtz K, He F, Sun X, Thomas RG *et al*: **A phase 3 trial of semagacestat for treatment of Alzheimer's disease**. *N Engl J Med* 2013, **369**(4):341-350.

21. Edmans J, Bradshaw L, Franklin M, Gladman J, Conroy S: **Specialist geriatric medical assessment for patients discharged from hospital acute assessment units: randomised controlled trial**. *Bmj* 2013, **347**:f5874.

22. Engebretson SP, Hyman LG, Michalowicz BS, Schoenfeld ER, Gelato MC, Hou W, Seaquist ER, Reddy MS, Lewis CE, Oates TW *et al*: **The effect of nonsurgical periodontal therapy on hemoglobin A1c levels in persons with type 2 diabetes and chronic periodontitis: a randomized clinical trial**. *Jama* 2013, **310**(23):2523-2532.

23. Feagan BG, Rutgeerts P, Sands BE, Hanauer S, Colombel JF, Sandborn WJ, Van Assche G, Axler J, Kim HJ, Danese S *et al*: **Vedolizumab as induction and maintenance therapy for ulcerative colitis**. *N Engl J Med* 2013, **369**(8):699-710.

24. Finkelstein JS, Lee H, Burnett-Bowie SA, Pallais JC, Yu EW, Borges LF, Jones BF, Barry CV, Wulczyn KE, Thomas BJ *et al*: **Gonadal steroids and body composition, strength, and sexual function in men**. *N Engl J Med* 2013, **369**(11):1011-1022.

25. Foa EB, McLean CP, Capaldi S, Rosenfield D: **Prolonged exposure vs supportive counseling for sexual abuse-related PTSD in adolescent girls: a randomized clinical trial**. *Jama* 2013, **310**(24):2650-2657.

26. Foa EB, Yusko DA, McLean CP, Suvak MK, Bux DA, Jr., Oslin D, O'Brien CP, Imms P, Riggs DS, Volpicelli J: **Concurrent naltrexone and prolonged exposure therapy for patients with comorbid alcohol dependence and PTSD: a randomized clinical trial**. *Jama* 2013, **310**(5):488-495.

27. Ghofrani HA, D'Armini AM, Grimminger F, Hoeper MM, Jansa P, Kim NH, Mayer E, Simonneau G, Wilkins MR, Fritsch A *et al*: **Riociguat for the treatment of chronic thromboembolic pulmonary hypertension**. *N Engl J Med* 2013, **369**(4):319-329.

28. Ghofrani HA, Galie N, Grimminger F, Grunig E, Humbert M, Jing ZC, Keogh AM, Langleben D, Kilama MO, Fritsch A *et al*: **Riociguat for the treatment of pulmonary arterial hypertension**. *N Engl J Med* 2013, **369**(4):330-340.

29. Goldberg SE, Bradshaw LE, Kearney FC, Russell C, Whittamore KH, Foster PE, Mamza J, Gladman JR, Jones RG, Lewis SA *et al*: **Care in specialist medical and mental health unit compared with standard care for older people with cognitive impairment admitted to general hospital: randomised controlled trial (NIHR TEAM trial)**. *Bmj* 2013, **347**:f4132.

30. Harris DL, Weston PJ, Signal M, Chase JG, Harding JE: **Dextrose gel for neonatal hypoglycaemia (the Sugar Babies Study): a randomised, double-blind, placebo-controlled trial**. *Lancet* 2013, **382**(9910):2077-2083.

31. Kelleher J, Bhat R, Salas AA, Addis D, Mills EC, Mallick H, Tripathi A, Pruitt EP, Roane C, McNair T *et al*: **Oronasopharyngeal suction versus wiping of the mouth and nose at birth: a randomised equivalency trial**. *Lancet* 2013, **382**(9889):326-330.

32. Kimmel SE, French B, Kasner SE, Johnson JA, Anderson JL, Gage BF, Rosenberg YD, Eby CS, Madigan RA, McBane RB *et al*: **A pharmacogenetic versus a clinical algorithm for warfarin dosing**. *N Engl J Med* 2013, **369**(24):2283-2293.

33. Knapp M, King D, Romeo R, Schehl B, Barber J, Griffin M, Rapaport P, Livingston D, Mummery C, Walker Z *et al*: **Cost effectiveness of a manual based coping strategy programme in promoting the mental health of family carers of people with dementia (the START (STrAtegies for RelaTives) study): a pragmatic randomised controlled trial**. *Bmj* 2013, **347**:f6342.

34. Kolle SF, Fischer-Nielsen A, Mathiasen AB, Elberg JJ, Oliveri RS, Glovinski PV, Kastrup J, Kirchhoff M, Rasmussen BS, Talman ML *et al*: **Enrichment of autologous fat grafts with ex-vivo expanded adipose tissue-derived stem cells for graft survival: a randomised placebo-controlled trial**. *Lancet* 2013, **382**(9898):1113-1120.

35. Kravitz RL, Franks P, Feldman MD, Tancredi DJ, Slee CA, Epstein RM, Duberstein PR, Bell RA, Jackson-Triche M, Paterniti DA *et al*: **Patient engagement programs for recognition and initial treatment of depression in primary care: a randomized trial**. *Jama* 2013, **310**(17):1818-1828.

36. Labrie J, Berghmans BL, Fischer K, Milani AL, van der Wijk I, Smalbraak DJ, Vollebregt A, Schellart RP, Graziosi GC, van der Ploeg JM *et al*: **Surgery versus physiotherapy for stress urinary incontinence**. *N Engl J Med* 2013, **369**(12):1124-1133.

37. Lazzerini M, Martelossi S, Magazzu G, Pellegrino S, Lucanto MC, Barabino A, Calvi A, Arrigo S, Lionetti P, Lorusso M *et al*: **Effect of thalidomide on clinical remission in children and adolescents with refractory Crohn disease: a randomized clinical trial**. *Jama* 2013, **310**(20):2164-2173.

38. Ledwidge M, Gallagher J, Conlon C, Tallon E, O'Connell E, Dawkins I, Watson C, O'Hanlon R, Bermingham M, Patle A *et al*: **Natriuretic peptide-based screening and collaborative care for heart failure: the STOP-HF randomized trial**. *Jama* 2013, **310**(1):66-74.

39. Liem S, Schuit E, Hegeman M, Bais J, de Boer K, Bloemenkamp K, Brons J, Duvekot H, Bijvank BN, Franssen M *et al*: **Cervical pessaries for prevention of preterm birth in women with a multiple pregnancy (ProTWIN): a multicentre, open-label randomised controlled trial**. *Lancet* 2013, **382**(9901):1341-1349.

40. Little P, Hobbs FD, Moore M, Mant D, Williamson I, McNulty C, Cheng YE, Leydon G, McManus R, Kelly J *et al*: **Clinical score and rapid antigen detection test to guide antibiotic use for sore throats: randomised controlled trial of PRISM (primary care streptococcal management)**. *Bmj* 2013, **347**:f5806.

41. Little P, Moore M, Kelly J, Williamson I, Leydon G, McDermott L, Mullee M, Stuart B: **Ibuprofen, paracetamol, and steam for patients with respiratory tract infections in primary care: pragmatic randomised factorial trial**. *Bmj* 2013, **347**:f6041.

42. Livingston G, Barber J, Rapaport P, Knapp M, Griffin M, King D, Livingston D, Mummery C, Walker Z, Hoe J *et al*: **Clinical effectiveness of a manual based coping strategy programme (START, STrAtegies for RelaTives) in promoting the mental health of carers of family members with dementia: pragmatic randomised controlled trial**. *Bmj* 2013, **347**:f6276.

43. Ly TT, Nicholas JA, Retterath A, Lim EM, Davis EA, Jones TW: **Effect of sensor-augmented insulin pump therapy and automated insulin suspension vs standard insulin pump therapy on hypoglycemia in patients with type 1 diabetes: a randomized clinical trial**. *Jama* 2013, **310**(12):1240-1247.

44. Manley BJ, Owen LS, Doyle LW, Andersen CC, Cartwright DW, Pritchard MA, Donath SM, Davis PG: **High-flow nasal cannulae in very preterm infants after extubation**. *N Engl J Med* 2013, **369**(15):1425-1433.

45. Martinez-Garcia MA, Capote F, Campos-Rodriguez F, Lloberes P, Diaz de Atauri MJ, Somoza M, Masa JF, Gonzalez M, Sacristan L, Barbe F *et al*: **Effect of CPAP on blood pressure in patients with obstructive sleep apnea and resistant hypertension: the HIPARCO randomized clinical trial**. *Jama* 2013, **310**(22):2407-2415.

46. Marty FM, Winston DJ, Rowley SD, Vance E, Papanicolaou GA, Mullane KM, Brundage TM, Robertson AT, Godkin S, Mommeja-Marin H *et al*: **CMX001 to prevent cytomegalovirus disease in hematopoietic-cell transplantation**. *N Engl J Med* 2013, **369**(13):1227-1236.

47. McDermott MM, Liu K, Guralnik JM, Criqui MH, Spring B, Tian L, Domanchuk K, Ferrucci L, Lloyd-Jones D, Kibbe M *et al*: **Home-based walking exercise intervention in peripheral artery disease: a randomized clinical trial**. *Jama* 2013, **310**(1):57-65.

48. McInnes IB, Kavanaugh A, Gottlieb AB, Puig L, Rahman P, Ritchlin C, Brodmerkel C, Li S, Wang Y, Mendelsohn AM *et al*: **Efficacy and safety of ustekinumab in patients with active psoriatic arthritis: 1 year results of the phase 3, multicentre, double-blind, placebo-controlled PSUMMIT 1 trial**. *Lancet* 2013, **382**(9894):780-789.

49. Mendelow AD, Gregson BA, Rowan EN, Murray GD, Gholkar A, Mitchell PM: **Early surgery versus initial conservative treatment in patients with spontaneous supratentorial lobar intracerebral haematomas (STICH II): a randomised trial**. *Lancet* 2013, **382**(9890):397-408.

50. Mentzelopoulos SD, Malachias S, Chamos C, Konstantopoulos D, Ntaidou T, Papastylianou A, Kolliantzaki I, Theodoridi M, Ischaki H, Makris D *et al*: **Vasopressin, steroids, and epinephrine and neurologically favorable survival after in-hospital cardiac arrest: a randomized clinical trial**. *Jama* 2013, **310**(3):270-279.

51. Messier SP, Mihalko SL, Legault C, Miller GD, Nicklas BJ, DeVita P, Beavers DP, Hunter DJ, Lyles MF, Eckstein F *et al*: **Effects of intensive diet and exercise on knee joint loads, inflammation, and clinical outcomes among overweight and obese adults with knee osteoarthritis: the IDEA randomized clinical trial**. *Jama* 2013, **310**(12):1263-1273.

52. Metsch LR, Feaster DJ, Gooden L, Schackman BR, Matheson T, Das M, Golden MR, Huffaker S, Haynes LF, Tross S *et al*: **Effect of risk-reduction counseling with rapid HIV testing on risk of acquiring sexually transmitted infections: the AWARE randomized clinical trial**. *Jama* 2013, **310**(16):1701-1710.

53. Moojen WA, Arts MP, Jacobs WC, van Zwet EW, van den Akker-van Marle ME, Koes BW, Vleggeert-Lankamp CL, Peul WC: **Interspinous process device versus standard conventional surgical decompression for lumbar spinal stenosis: randomized controlled trial**. *Bmj* 2013, **347**:f6415.

54. Morelli A, Ertmer C, Westphal M, Rehberg S, Kampmeier T, Ligges S, Orecchioni A, D'Egidio A, D'Ippoliti F, Raffone C *et al*: **Effect of heart rate control with esmolol on hemodynamic and clinical outcomes in patients with septic shock: a randomized clinical trial**. *Jama* 2013, **310**(16):1683-1691.

55. Mourvillier B, Tubach F, van de Beek D, Garot D, Pichon N, Georges H, Lefevre LM, Bollaert PE, Boulain T, Luis D *et al*: **Induced hypothermia in severe bacterial meningitis: a randomized clinical trial**. *Jama* 2013, **310**(20):2174-2183.

56. Nicholls SJ, Bakris GL, Kastelein JJ, Menon V, Williams B, Armbrecht J, Brunel P, Nicolaides M, Hsu A, Hu B *et al*: **Effect of aliskiren on progression of coronary disease in patients with prehypertension: the AQUARIUS randomized clinical trial**. *Jama* 2013, **310**(11):1135-1144.

57. O'Dell JR, Mikuls TR, Taylor TH, Ahluwalia V, Brophy M, Warren SR, Lew RA, Cannella AC, Kunkel G, Phibbs CS *et al*: **Therapies for active rheumatoid arthritis after methotrexate failure**. *N Engl J Med* 2013, **369**(4):307-318.

58. Osinusi A, Meissner EG, Lee YJ, Bon D, Heytens L, Nelson A, Sneller M, Kohli A, Barrett L, Proschan M *et al*: **Sofosbuvir and ribavirin for hepatitis C genotype 1 in patients with unfavorable treatment characteristics: a randomized clinical trial**. *Jama* 2013, **310**(8):804-811.

59. Parkman HP, Van Natta ML, Abell TL, McCallum RW, Sarosiek I, Nguyen L, Snape WJ, Koch KL, Hasler WL, Farrugia G *et al*: **Effect of nortriptyline on symptoms of idiopathic gastroparesis: the NORIG randomized clinical trial**. *Jama* 2013, **310**(24):2640-2649.

60. Pirmohamed M, Burnside G, Eriksson N, Jorgensen AL, Toh CH, Nicholson T, Kesteven P, Christersson C, Wahlstrom B, Stafberg C *et al*: **A randomized trial of genotype-guided dosing of warfarin**. *N Engl J Med* 2013, **369**(24):2294-2303.

61. Poole J, Mavromatis K, Binongo JN, Khan A, Li Q, Khayata M, Rocco E, Topel M, Zhang X, Brown C *et al*: **Effect of progenitor cell mobilization with granulocyte-macrophage colony-stimulating factor in patients with peripheral artery disease: a randomized clinical trial**. *Jama* 2013, **310**(24):2631-2639.

62. Powers SW, Kashikar-Zuck SM, Allen JR, LeCates SL, Slater SK, Zafar M, Kabbouche MA, O'Brien HL, Shenk CE, Rausch JR *et al*: **Cognitive behavioral therapy plus amitriptyline for chronic migraine in children and adolescents: a randomized clinical trial**. *Jama* 2013, **310**(24):2622-2630.

63. Saitz R, Cheng DM, Winter M, Kim TW, Meli SM, Allensworth-Davies D, Lloyd-Travaglini CA, Samet JH: **Chronic care management for dependence on alcohol and other drugs: the AHEAD randomized trial**. *Jama* 2013, **310**(11):1156-1167.

64. Sandborn WJ, Feagan BG, Rutgeerts P, Hanauer S, Colombel JF, Sands BE, Lukas M, Fedorak RN, Lee S, Bressler B *et al*: **Vedolizumab as induction and maintenance therapy for Crohn's disease**. *N Engl J Med* 2013, **369**(8):711-721.

65. Sihvonen R, Paavola M, Malmivaara A, Itala A, Joukainen A, Nurmi H, Kalske J, Jarvinen TL: **Arthroscopic partial meniscectomy versus sham surgery for a degenerative meniscal tear**. *N Engl J Med* 2013, **369**(26):2515-2524.

66. Specks U, Merkel PA, Seo P, Spiera R, Langford CA, Hoffman GS, Kallenberg CG, St Clair EW, Fessler BJ, Ding L *et al*: **Efficacy of remission-induction regimens for ANCA-associated vasculitis**. *N Engl J Med* 2013, **369**(5):417-427.

67. Spijkerman J, Veenhoven RH, Wijmenga-Monsuur AJ, Elberse KE, van Gageldonk PG, Knol MJ, de Melker HE, Sanders EA, Schouls LM, Berbers GA: **Immunogenicity of 13-valent pneumococcal conjugate vaccine administered according to 4 different primary immunization schedules in infants: a randomized clinical trial**. *Jama* 2013, **310**(9):930-937.

68. Strain WD, Lukashevich V, Kothny W, Hoellinger MJ, Paldanius PM: **Individualised treatment targets for elderly patients with type 2 diabetes using vildagliptin add-on or lone therapy (INTERVAL): a 24 week, randomised, double-blind, placebo-controlled study**. *Lancet* 2013, **382**(9890):409-416.

69. Thielmann M, Kottenberg E, Kleinbongard P, Wendt D, Gedik N, Pasa S, Price V, Tsagakis K, Neuhauser M, Peters J *et al*: **Cardioprotective and prognostic effects of remote ischaemic preconditioning in patients undergoing coronary artery bypass surgery: a single-centre randomised, double-blind, controlled trial**. *Lancet* 2013, **382**(9892):597-604.

70. Thom S, Poulter N, Field J, Patel A, Prabhakaran D, Stanton A, Grobbee DE, Bots ML, Reddy KS, Cidambi R *et al*: **Effects of a fixed-dose combination strategy on adherence and risk factors in patients with or at high risk of CVD: the UMPIRE randomized clinical trial**. *Jama* 2013, **310**(9):918-929.

71. Tracy SK, Hartz DL, Tracy MB, Allen J, Forti A, Hall B, White J, Lainchbury A, Stapleton H, Beckmann M *et al*: **Caseload midwifery care versus standard maternity care for women of any risk: M@NGO, a randomised controlled trial**. *Lancet* 2013, **382**(9906):1723-1732.

72. Tsai JN, Uihlein AV, Lee H, Kumbhani R, Siwila-Sackman E, McKay EA, Burnett-Bowie SA, Neer RM, Leder BZ: **Teriparatide and denosumab, alone or combined, in women with postmenopausal osteoporosis: the DATA study randomised trial**. *Lancet* 2013, **382**(9886):50-56.

73. Tsujinaka T, Yamamoto K, Fujita J, Endo S, Kawada J, Nakahira S, Shimokawa T, Kobayashi S, Yamasaki M, Akamaru Y *et al*: **Subcuticular sutures versus staples for skin closure after open gastrointestinal surgery: a phase 3, multicentre, open-label, randomised controlled trial**. *Lancet* 2013, **382**(9898):1105-1112.

74. Verhoef TI, Ragia G, de Boer A, Barallon R, Kolovou G, Kolovou V, Konstantinides S, Le Cessie S, Maltezos E, van der Meer FJ *et al*: **A randomized trial of genotype-guided dosing of acenocoumarol and phenprocoumon**. *N Engl J Med* 2013, **369**(24):2304-2312.

75. Walmsley SL, Antela A, Clumeck N, Duiculescu D, Eberhard A, Gutierrez F, Hocqueloux L, Maggiolo F, Sandkovsky U, Granier C *et al*: **Dolutegravir plus abacavir-lamivudine for the treatment of HIV-1 infection**. *N Engl J Med* 2013, **369**(19):1807-1818.

76. Weinstein SL, Dolan LA, Wright JG, Dobbs MB: **Effects of bracing in adolescents with idiopathic scoliosis**. *N Engl J Med* 2013, **369**(16):1512-1521.

77. Zeuzem S, Soriano V, Asselah T, Bronowicki JP, Lohse AW, Mullhaupt B, Schuchmann M, Bourliere M, Buti M, Roberts SK *et al*: **Faldaprevir and deleobuvir for HCV genotype 1 infection**. *N Engl J Med* 2013, **369**(7):630-639.
